# Pillared graphene as an ultra-high sensitivity mass sensor

**DOI:** 10.1038/s41598-017-14182-6

**Published:** 2017-10-25

**Authors:** Ke Duan, Li Li, Yujin Hu, Xuelin Wang

**Affiliations:** State Key Laboratory of Digital Manufacturing Equipment and Technology, School of Mechanical Science and Engineering, Huazhong University of Science and Technology, Wuhan, 430074 China

## Abstract

Hybrid structure of graphene sheets supported by carbon nanotubes (CNTs) sustains unique properties of both graphene and CNTs, which enables the utilization of advantages of the two novel materials. In this work, the capability of three-dimensional pillared graphene structure used as nanomechanical sensors is investigated by performing molecular dynamics simulations. The obtained results demonstrate that: (a) the mass sensitivity of the pillared graphene structure is ultrahigh and can reach at least 1 yg (10^−24^ g) with a mass responsivity 0.34 GHz · yg^−1^; (b) the sizes of pillared graphene structure, particularly the distance between carbon nanotube pillars, have a significant effect on the sensing performance; (c) an analytical expression can be derived to detect the deposited mass from the resonant frequency of the pillared graphene structure. The performed analyses might be significant to future design and application of pillared graphene based sensors with high sensitivity and large detecting area.

## Introduction

Advances in micro- and nano-materials synthesis have significantly motivated the development of a variety of new nano-technologies, including nanoelectrochemical systems^1^, resonance based nanosensors^2–7^, and nanoactuators^8^. In particular, nanoscale sensors have received tremendous attentions over the past few years owing to their extreme mass and force sensitivity, and play crucial roles in numerous advanced technological applications, with select examples including mass detection^9–11^ (such as detection of gas and biological molecules), chemical process control^12^, and basic phenomena in surface science^13,14^ (such as transitions and diffusion). Building and designing such nanosensors that not only able to effective capture the incoming mass flux but also capable of detecting smaller attached masses is the ultimate aim of any mass sensing techniques.

Any materials or structures that can achieve very high resonant frequency as well as very low density are appreciated to design the high performance sensors. The sensitivity of a nanomechanical mass sensor can be defined as the minimum mass value needed to induce a specified frequency shift in the sensor. The minimum detectable mass, *δm*, can be expressed as *δm* = (2*m*_*eff*_/*f*_0_)*δf*_0_^15^. Among the given formula, *m*_*eff*_ is the effective mass of sensors, *f*_0_ represents the resonance frequency of the resonator and *δf*_0_ is the standard deviation of the measured frequency. Here, the quantity (2*m*_*eff*_/*f*_0_)^−1^ represents the mass responsivity, which is a significant parameter to evaluate the sensing performance of the resonance based sensors. It is clear that a high mass responsivity is appreciated to achieve a high sensing resolution. Therefore, resonators with low mass and high resonance frequency are expected to exhibit extraordinary sensitivity.

Over the past decade, graphene and carbon nanotubes (CNTs) have attracted significant attentions in the field of high performance nanomechanical sensors owing to their exceptional mechanical property and low mass density. CNT-based resonators can achieve high frequency shifts even a very small external mass was adsorbed onto their surfaces and therefore show outstanding mass sensitivity^10,16–18^. For example, Lassagne *et al*. reported a mass resolution of 1.4 zg (1 zg = 10^−21^ g) at temperature 5 K using a 1 nm diameter carbon nanotube as the nanomechanical mass sensor^18^. When decreasing the dimensions of carbon nanotube, the resonator could even detecting a mass as low as ~200 yg (1 yg = 10^−24^ g)^3,10,19^. Compared with carbon nanotube, graphene-based nanomechanical sensors show a larger minimum detectable mass (an order of 10^−21^ g) characteristics even operated at cryogenic temperature^20–23^. However, graphene possesses an important advantage over carbon nanotubes that its platelet structure provides a larger exposed surface area for interaction with its surroundings and makes it very efficient for detecting the incoming mass flux. Undoubtedly, an ideal mass nanosensors requires both high mass resolution and a larger detecting surface area. Recently, many efforts have been paid to fabricate more advanced 3D carbon nanostructure materials by combining carbon nanotubes and graphene sheets^24–26^. One of such novel carbon structures is a pillared graphene structures supported by vertically aligned CNTs (VACNT)^27–30^. The superior multi-dimensional functionality of the VACNT-graphene structure make its applications possible in many potential applications such as energy storage and gas separation membranes^31,32^. From the mass nanosensor point of view, the VACNT-graphene nanostructure may combines the advantages of both CNTs (high sensitivity) and graphene (larger detecting surface area), and hence exhibits extraordinary performance in mass sensing.

Molecular dynamics (MD) simulation method seems to be the most suitable way compared with other methods such as nonlocal elasticity theory or nano-experiment method to explore the capability of the pillared-graphene structure as a sensor due to its complex geometry configuration. Therefore, in this work, we employ MD to examine the possibility and capability of VACNT-graphene nanostructure used as a mass resonator. The effects of attached mass location and geometric parameters of the nanostructure, including the inter-pillar distance and pillar height, are analyzed. The specific relationships between resonant frequency of the studied structure and the attached mass are determined.

## Simulation Details

**Figure 1:**
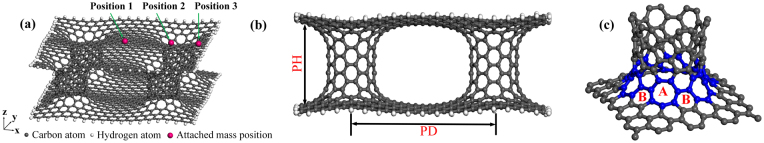
(**a**) Schematic of the VACNT-graphene structure with different attached mass location. (**b**) Front view. (**c**) Junction of graphene sheet and a (6, 6) armchair CNT. Atoms at the joint are marked as blue and type A represents the heptagon rings while type B denotes the hexagon rings.

According to the research work of Matsumoto and Saito, varies of shapes may formed with the graphene sheets and vertically aligned carbon nanotubes^33^. In this work, the structure constituted of two graphene sheets and four carbon nanotube pillars was considered and its minimum energy state is shown in Fig. 1(a). This structure was constructed by creating cylindrical holes on the graphene sheets and connecting the two sheets with four vertically aligned (6, 6) armchair CNTs. By rotating the nanotube, axially symmetric heptagonal and hexagonal rings at the joint can be formed, as depicted in Fig. 1(c). The used graphene sheets were square-shaped and the distance between two nearest nanotubes was identical. The size of graphene sheets and the length of carbon nanotubes can be altered to construct a specified dimensions system. To differentiate these systems that have different dimensions, the term “PHhPDd” was utilized in this study, where “PHh” denotes the height of tubes (h), and “PDd” represents the distance (d) between vertically aligned nanotubes^30^. Furthermore, it is noteworthy that the edge carbon atoms were passivated with hydrogen atoms in order to suppress its spurious edge modes; recent works has validated that hydrogen passivation method is effective in stabilizing the free edge graphene atoms and hence leaving the edges essentially stress-free^34,35^.

In this work, we utilized the Adaptive Intermolecular Reactive Empirical Bond Order (AIREBO) potential for both carbon-carbon and carbon-hydrogen interactions; this potential has been demonstrated to accurately predict the binding energy and elastic properties as well as dynamic characteristics of a series of carbon-based nanomaterials^36–38^. Similar to most of the existing works, the materials deposited on the graphene sheet were assumed to be a “point mass” and its mass were distributed equally on carbon atoms (equivalent mass atoms) near the deposited position^39,40^. In addition, we assume that the VACNT-graphene structure was tightly connected to its substrate and hence the bottom graphene sheet was fixed in our simulations. It appears to be reasonable to use the fixed condition as an analog of interaction with the most widely used silicon or silicon-oxide substrate due to the strong adhesion between graphene sheet and the substrate^41–43^. Based on the above two assumptions, we now provide the details of investigating the resonant frequency of VACNT-graphene structure.

### Equilibration

For all simulations, the VACNT-graphene structure was first optimized using a conjugate gradient algorithm by adjusting their position to obtain a much more reasonable atomic arrangement. Subsequently, the free boundary conditions were applied, and the optimized structure was further equilibrated at a constant temperature 10 K using a Nose-Hoover thermostat for 200 ps with a time step 1 fs under a NVT ensemble [where the number of atoms (N), volume (V), and temperature (T) are kept constant]. Then, the bottom layer of the graphene sheet was fixed and the system was equilibrated again for 200 ps.

### Excitation and vibration

After the equilibration process, a force along the positive z-axis direction was applied on the equivalent mass atoms to induce a small displacement. Then, the applied force was removed and the structure was allowed to oscillate freely in a micro-canonical form (NVE) where the total energy of the system is conserved. The increase in total energy of the system induced by the applied force was less than 0.03% of the total potential energy of the system to ensure that the nonlinear vibration modes would not be excited.

The vibration response of the attached mass atom is extracted for analysing the fundamental frequency of the VACNT-graphene structure by using the Fast Fourier Transform (FFT) method. All of the performed molecular dynamics simulations were carried out by the Large-scale Atomic/Molecular Massively Parallel Simulator (LAMMPS) package^44^, while the OVITO package was utilized for visualization^45^.

## Results and Discussion

### Effects of attached mass location

Nanoscale mass resonator is based on the fact that resonant frequency shifts when an external mass is attached. The key issue of mass detection is the measurement of change in resonant frequency due to the deposited mass. In general, the shifts in the resonant frequency of an atomic-scale sensor are directly related with the location of the added mass, geometric parameters of the structure, and values of the attached mass. In this section, a VACNT-graphene structure consists of two graphene sheets and four CNT pillars was used to investigate the effects of attached mass location on the mass sensitivity of the structure. The used structure had dimensions of PD27PH14 (d = 27 Å, h = 14 Å). Three typical attached mass locations were considered, i.e., position 1: at the center of upper sheet, position 2: on the upper sheet and at the center place of two pillars, and position 3: at the middle place of the suspended edge of the upper sheet, as shown in Fig. 1(a).

**Figure 2:**
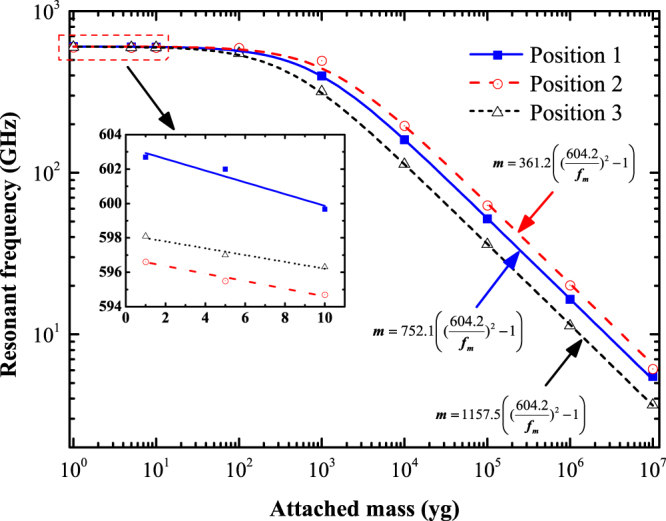
Effect of position of the attached mass on the fundamental resonant frequency of PD27PH14.

Figure 2 shows the variation in resonant frequency of the PD27PH14 with the added mass. As observed in Fig. 2, the resonant frequency decreases with the increase in attached mass no matter which location of the added mass is. In particular, the dependence of resonant frequency on the attached mass follows a specific law that can be expressed as follows:

1 $$m=\alpha \,({(\tfrac{{f}_{0}}{{f}_{m}})}^{2}-1)$$

where *m* stands for the attached mass, *f*_0_ and *f*_*m*_ denote the resonant frequency of resonator without and with attached mass, and *α* is constant obtained using the least-squares fit from the simulation results. It is noteworthy that the calculated fundamental resonant frequency (*f*_0_) of the PD27PH14 without mass attached is 604.2 GHz. The calculated law for the three different location cases are presented in Fig. 2. Furthermore, the inset in Fig. 2 shows the resonant frequency as a function of deposited mass that are varied from 1 yg to 10 yg. The slopes of the fitting curves in the inset give the value of mass responsivity for the structure PD27PH14 at different locations. The obtained mass responsivity are 0.34, 0.2, and 0.21 GHz · yg^−1^ for the position 1 to position 3, respectively. This is seven orders of magnitude better than the previously reported work about carbon nanotube based mass sensors^18^. This extraordinary mass responsivity may attributed to the extremely low mass of the structure and the vacuum simulation condition without considering any external factors.

**Figure 3:**
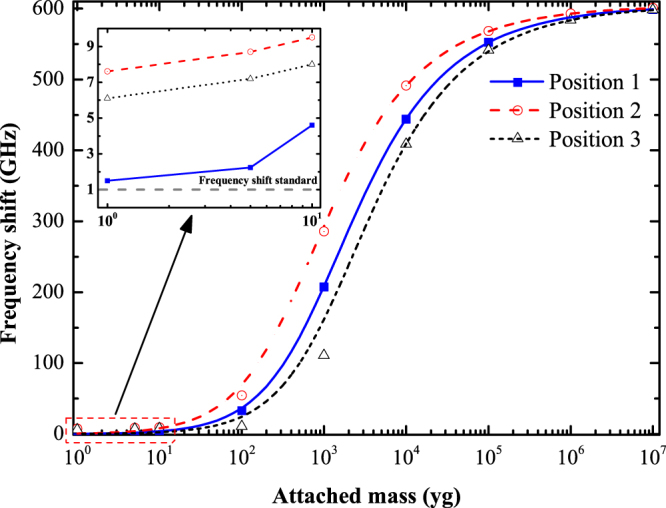
Resonant frequency shifts of PD27PH14 versus attached mass.

Having demonstrated the inherent law of resonant frequency versus external mass and the high responsivity of the pillared-graphene structure, we now determine the mass sensitivity. As previously mentioned, the mass sensitivity of a mass sensor is defined as the minimum value needed to reflect a specified frequency shift. In this work, the sensitivity of the pillared graphene structure is deemed as the needed attached mass value to give a frequency shift of approximately 1 GHz (frequency shift standard), which is a somewhat tougher standard than some other open literatures^39,46^. Figure 3 shows the variation in resonant frequency shifts for the mass-attached pillared-graphene structure, where the frequency shift is defined as (Δ*f* = *f*_0_ − *f*_*m*_). The inset shows the enlarged view of the situations where attached mass ranges from 1 yg to 10 yg. The gray dash line in the inset represents the frequency shift standard (1 GHz). It is clear that the frequency shift exceeds 1 GHz for the three positions when 1 yg external mass was deposited.

To better clarify the mass sensitivity, we list in Table 1 the frequency shift of PD27PH14 that attached with varies mass at different locations. As seen in Table 1, the minimum shift in resonant frequency of PD27PH14 with a 1 yg attached mass is 1.5 GHz occurred at the position 1. Clearly, the sensitivity of PD27PH14 could reach at least 1 yg (10^−24^ g), which is much higher than that of graphene based mass sensor reported in open literatures^22,40,47^. The sensing performance of the studied structure even exceeds the single-walled carbon nanotube based mass sensors (10^3^ yg)^16^. The observed extraordinary sensing performance of PD27PH14 may induced by the arch shaped graphene sheet, which is clearly depicted in Fig. 1(b). The stiffness of the arch shaped graphene sheet is much larger compared to its platelet 2D shape and hence show a better sensing capability; the stiffness enhancing mechanism is very similar to the practical applications that bending the sheet metal can significantly enhance their stiffness. Therefore, the VACNT-graphene structure can act as an outstanding mass sensor that not only possess a larger detecting surface area but also show ultrahigh mass sensitivity.

**Table 1:** The resonant frequency shift (GHz) of VACNT-graphene structure that attached with varies mass (yg) at different positions.

Mass	1	5	10^1^	10^2^	10^3^	10^4^	10^5^	10^6^	10^7^
Position 1	1.5	2.2	4.5	32.9	207.5	444.3	552.3	587.7	598.7
Position 2	6.1	7.2	8.0	10.9	111.0	408.9	541.3	584.1	598.1
Position 3	7.6	8.7	9.5	54.6	285.6	491.3	568.2	592.9	600.5

### Effects of geometric parameters

The sensing capability of a nanomechanical resonator has been demonstrated to directly related to their dimensions. For instance, the mass sensitivity of a 40-nm-long CNT resonator could reaches few tens of yg while a 200-nm-long CNT resonator could only detects zg (10^−21^ *g*) attached masses^46^. Therefore, it is necessary to determine the effects of geometric parameters of the studied VACNT-graphene structure on its mass sensitivity. In this section, two key geometric parameters, including PD and PH, are considered.

#### Determining the influence of PD

**Figure 4:**
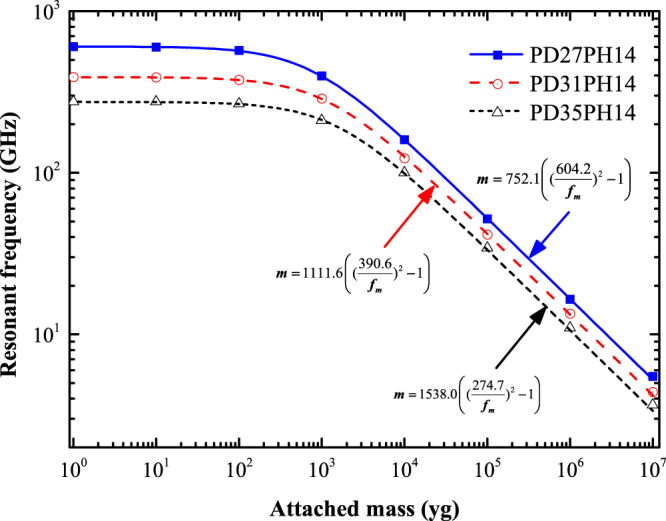
Fundamental resonant frequency as a function of the attached mass for VACNT-graphene structures with different PD.

Three different types of VACNT-graphene structures were considered to study the PD dependence of the resonant frequency, each of them having a tube height of 14 Å, and with PD as 27 Å, 31 Å, and 35 Å. According to the pre-mentioned terminology, the three different structures are termed as PD27PH14, PD31PH14, and PD35PH14, respectively. For all the following simulations, the added mass was attached on the middle of the upper sheet, i.e., only the position 1 was considered. Figure 4 shows the variation of resonant frequency with the attached mass for these three kinds of VACNT-graphene structures. For all sizes, the resonant frequency is found to decrease with the increase in attached mass. In addition, from the the fitted expression presented in Fig. 4, we can conclude that the resonant frequency of no-mass attached VACNT-graphene structure decreases with the increases in geometric parameter PD, where the calculated fundamental frequency of the three kinds of structures: PD27PH14, PD31PH14, and PD35PH14 are 604.2 GHz, 390.6 GHz, and 274.7 GHz, respectively. However, the coefficient *α* increases with the increases in PD and are 752.1, 879.5, and 1203.2, respectively for structures: PD27PH14, PD31PH14, and PD35PH14. For practical applications, the parameter *α* can be tabulated in terms of sizes of the VACNT-graphene structure.

**Figure 5:**
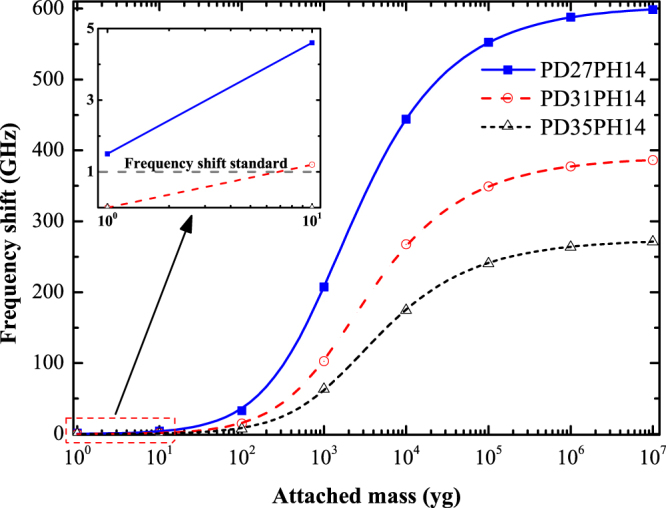
Resonant frequency shifts of VACNT-graphene structures with different PD versus attached mass.

Figure 5 shows the variation of frequency shift with the attached mass on the VACNT-graphene structures with different PD values. As seen from Fig. 5, the frequency shift is much larger for VACNT-graphene structure with a smaller PD when an identical external mass was attached. This phenomenon indicates that the mass sensing performance of the studied VACNT-graphene structure can be enhanced by reducing the value of PD. To further determine the effects of PD on the mass sensitivity of the VACNT-structure, we list in Table 2 the frequency shifts of the three types of structures with different attached mass. As can be seen in Table 2, for the case of PD31PH14, the frequency shift is zero when a 1 yg external mass was attached, which indicates that the sensitivity of PD31PH14 can only reach about 10 yg. Furthermore, the VACNT-graphene structure even could not detects a 10 yg attached mass if the PDd increases to 35 Å, as presented in Table 2 for the case of PD35PH14. Therefore, we can conclude that PD has significant effect on the mass sensitivity of the VACNT-graphene based nano-sensors. This characteristic enables the design of VACNT-graphene based nano-sensors with different sensitivity levels by controlling over the distance between pillars.

**Table 2:** The resonant frequency shift (GHz) of VACNT-graphene structures that attached with varies mass (yg).

Mass	1	10^1^	10^2^	10^3^	10^4^	10^5^	10^6^	10^7^
PD27PH14	1.5	4.5	32.9	207.5	444.3	552.3	587.7	598.7
PD31PH14	0.0	1.2	14.6	102.5	267.3	349.1	377.2	386.2
PD35PH14	0.0	0.0	7.3	63.5	174.6	240.5	263.7	280.0

#### Determining the influence of PH

**Figure 6:**
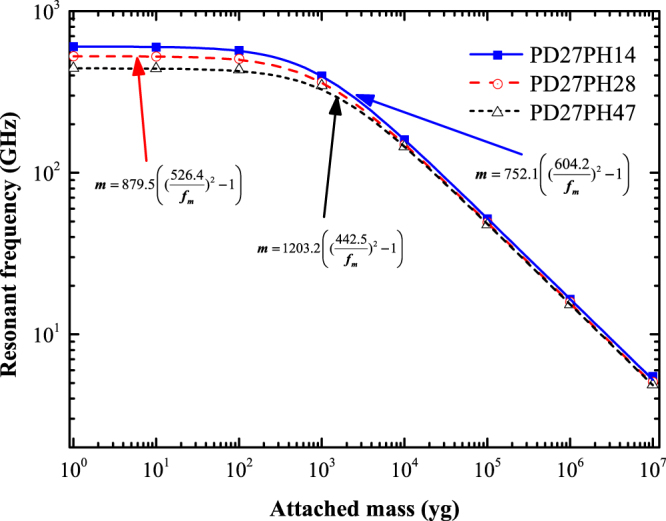
Fundamental resonant frequency as a function of the attached mass for VACNT-graphene structures with different PH.

To have a systematic understanding of the size dependence of the mass sensitivity of the studied VACNT-graphene structure, the impact of PH on mass sensing performance was also explored. Similar to the previous part, we also consider three cases for determining the influences of PH on mass sensitivity of the studied structure. The selected three structures are PD27PH14, PD27PH28, and PD27PH47, where the inter-pillar distance was identical and with tube height as 14 Å, 28 Å, and 47 Å. Figure 6 shows the variation of resonant frequency for these VACNT-graphene structures as a function of attached mass. As expected, the specific law (equation (1)) relationship is also observed. Moreover, the resonant frequency is found to decrease with the increase in tube height when a same external mass was attached. This phenomenon is reasonable and is attributed to the weaken stiffness that caused by the increased tube heights.

**Figure 7:**
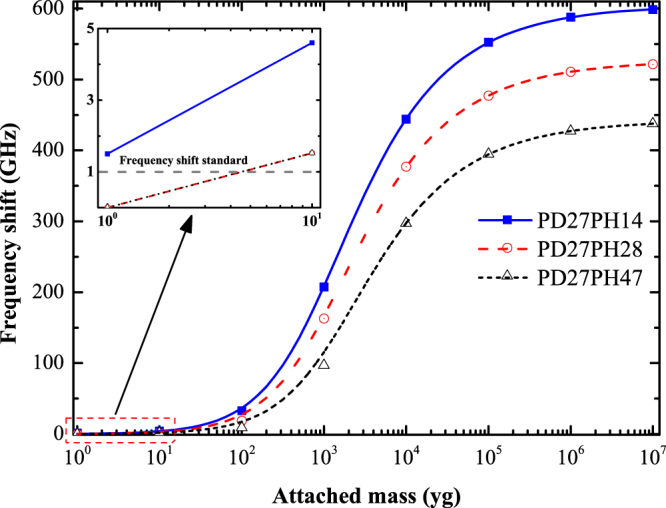
Resonant frequency shifts of VACNT-graphene structures with different PH versus attached mass.

For the studied three structures: PD27PH14, PD27PH28, and PD27PH47, the calculated fundamental frequency is 604.2 GHz, 526.4 GHz, and 442.5 GHz, respectively. Figure 7 shows the resonant frequency shift of the studied structures. It is clear that the structure with short tube height shows a larger shift in resonant frequency due to the higher stiffness. As we can seen from the inset in Fig. 7, there are no frequency shift for the cases of PD27PH28 and PD27PH47 when a 1 yg external mass was attached. When the deposited mass increased to 10 yg, the frequency shift increase to 1.5 GHz (shown in Table 3) which is greater than the frequency shift standard 1 GHz for the two structures. Therefore, the mass sensitivity is about 10 yg for the structures PD27PH28 and PD27PH47.

**Table 3:** The resonant frequency shift (GHz) of VACNT-graphene structures that attached with varies mass (yg).

Mass	1	10^1^	10^2^	10^3^	10^4^	10^5^	10^6^	10^7^
PD27PH14	1.5	4.5	32.9	207.5	444.3	552.3	587.7	598.7
PD27PH28	0.0	1.5	18.6	162.7	376.9	477.0	510.6	521.3
PD27PH47	0.0	1.5	9.2	97.0	297.2	394.9	427.2	437.6

According to the previous part, the mass sensitivity of the structure PD35PH14 that with a volume 42.6 *nm* ^3^ composed of 2380 carbon atoms and 188 hydrogen atoms is about tens of yg, as shown in Table 2. However, the sensitivity of the structure PD27PH28 that with a volume 96.9 *nm* ^3^ composed of 2260 carbon atoms and 156 hydrogen atoms can reach less than 10 yg, as can observed from Table 3. It is clear that the structure PD27PH28 has a much better sensing performance and a lower density than that of PD35PH14. The difference can be more pronounced if we compare the PD27PH47 with the structure PD35PH14. Therefore, we can conclude that PH has less effect on the mass sensitivity of the VACNT-graphene structure. This conclusion is of great significance for designing and fabricating the high performance VACNT-graphene based nanosensors. For instance, researchers can design devices that operate at different sensitivity levels by focusing on controlling the density of tubes instead of the growing speed of tubes along its longitudinal direction.

## Concluding

In summary, we investigated the capability of the pillared-graphene structure as a nanomechanical sensor by using molecular dynamics (MD) simulation method. The effects of attached mass location and two geometric parameters including the inter-pillar distance and tube height, are investigated. From the obtained results, the concluding remarks are drawn as follows:

- The mass sensitivity of the pillared graphene structure can reach at least 1 yg (10^−24^ g), which is even better than the carbon nanotube based nanosensors.
- The sensitivity of the pillared graphene structure is found to decline with the increase in inter-pillar distance (PD) or tube height (PH).
- The inter-pillar distance (PD) shows a much more significant effect on the mass sensitivity than that of tube height (PH).

With the advances in three-dimensional carbon nanotube graphene hybrid material synthesis technologies, we believe that our work can provide useful guidelines for designing the pillared-graphene based sensors that possess not only high sensitivity but also larger surface areas.
